# Parental Interference in Offspring’s Mate Choice: Sets of Actions and Counteractions Based on Both Perspectives

**DOI:** 10.1007/s10508-023-02544-3

**Published:** 2023-02-17

**Authors:** Anna Fišerová, Jan Havlíček, Marek Urban, Kamila Urban, Zuzana Štěrbová

**Affiliations:** 1 Faculty of Science, Charles University, Viničná 7, 128 00 Prague, Czech Republic; 2 Faculty of Humanities, Charles University, Prague, Czech Republic; 3 Faculty of Arts, Charles University, Prague, Czech Republic

**Keywords:** Parental influence, Mate choice, Manipulation, Manipulation tactics

## Abstract

Mate choice in humans is specific by the involvement of kin both in partner choice and in the functioning of the relationship. The influence of relatives ranges from providing advice all the way to arranged marriages. Existing research so far tended to map neither the actual parental interventions and further reactions (counteractions) nor the independently pursued behavior (actions) by which the offspring or their partners assert their interests. This study identified such sets of behaviors through semi-structured individual interviews with members of 20 dyads of adult offspring and their biological parents (five son–mother, five son–father, five daughter–mother, and five daughter–father dyads). Thematic analysis revealed sets of parental actions aimed at the offspring, their partners, and the couple as a whole, as well as further counteractions (reactions to parental interference) and independently pursued behavior (actions) of the offspring and their partners aimed at the parents in the context of the offspring’s previous or current relationship. Our findings showed that parental interference differed depending on the recipient: toward the couple were applied mainly indirect and supportive parental actions, whereas when facing their offspring or offspring’s partner, parents’ actions were more direct and disruptive. Our results thus indicate the importance of reporting about actual interactions which differ from the hypothetical settings used in prior studies by lower intensity. Moreover, by interviewing both members of dyads, we expanded the sets of actions and counteractions identified by prior studies and managed to limit self-reporting bias.

**Supplementary Information:**

The online version contains supplementary material available at 10.1007/s10508-023-02544-3.

## Introduction

In humans, relatives or other group members tend to be engaged in mate choice decisions (Apostolou, 2010; van den Berg et al., 2013). Active parental influence varies considerably across societies, ranging from relatively free choice, suggestions, and advice all the way to arranged marriages without consent (Bejanyan et al., 2015; Havlíček et al., 2022). Prior research indicates that parents also have a large impact on the couple’s well-being. Family’s approval of a relationship is associated with love, commitment, and other positive feelings toward the partner and with a higher relationship quality (Sinclair et al., 2015). Even approval from the partner’s parents reduces an individual’s relationship distress (Lee et al., 2010).

On a proximate level, it is more convenient for a family to get along with the offspring’s partner in order to maintain functioning family coalitions (Perilloux et al., 2011) or to strengthen social status via the offspring’s partner of the same or higher social class (Bates, 1942). Mate choice can have a significant impact on the whole family, because, for instance, if the partner leaves, parents will have to provide resources and care for their grandchild (Faulkner & Schaller, 2007). Partner preferences of the parents and the offspring do not always match (e.g., Buunk et al., 2008; Perilloux et al., 2011), which encourages the emergence of behaviors that help enforce or promote each party’s choices (Apostolou, 2015).

The manipulation tactics observed nowadays in relation to partner choice probably co-opt adaptations for manipulation which emerged in other domains as well as strategies that evolved to manipulate the offspring’s mating behavior (Apostolou et al., 2015). Both qualitative (Bates, 1942; Ikels, 1985; Sussman, 1953) and quantitative studies (e.g., Apostolou, 2013) show that in individualistic societies, parental influence often takes the form of “manipulation tactics” (Apostolou, 2007), both straightforward and less direct ones. In this paper, we try to avoid the term “manipulation”: it has negative connotations and we avoided it in our interviews because it could affect responses of the participants (Apostolou, 2013) and thereby also further analyses. We also do not use the term “tactics,” because it implies conscious, purposeful behavior.

An earlier study by Apostolou (2013) categorized parental actions (in the article called “tactics”) according to everyday parent–child (Buss, 1992) and dating couples’ mutual interactions (Buss et al., 1987), thus taking into account that some behavioral patterns it identified overlapped across contexts. In particular, this pertained to six parental actions: Coercion (e.g., yelling at the offspring), Hardball (e.g., forcing the offspring to do something by blackmailing), Reasoning (e.g., giving the offspring advice about romantic relationships), Monetary reward (here labeled “Carrot and stick”; e.g., stop giving the offspring money), the Silent treatment (e.g., not talking to the offspring when she/he does something I do not approve of), and Social comparison (e.g., compare the offspring’s behavior with someone else’s behavior). Other parental actions, namely Whom one should marry (e.g., give the offspring reasons why they should or should not date someone), Matchmaking (e.g., introduction of potential mates to the offspring), and Prevention (e.g., not allowing the offspring to dress provocatively), were identified in prior qualitative studies in the context of parent–offspring mate choice (Ikels, 1985; Sussman, 1953). And finally, actions Guilt trip (e.g., crying when the offspring does something the parent does not approve of), Chaperoning (e.g., frequent calls to check on the offspring), and the Use of relatives and friends (e.g., asking a relative or friend to try to influence the offspring to do something) seem unique to manipulation of offspring’s mate choices (Apostolou, 2013).

Interfering parental behavior also affects the offspring’s partner. Apostolou (2013) identified parental actions aimed at the offspring’s partner, such as Hardball (e.g., degrade the partner) and Monetary reward (which Apostolou calls “Lure”; e.g., promise and provide the potential partner with financial support, house, or money upon marrying the offspring) which were categorized according to previously identified parental actions that occur within the context of everyday parent–child interactions (e.g., Buss, 1992). In contrast, actions We are family (e.g., make the partner feel at home), and Dirty laundry (e.g., spy on the partner) were identified as unique to manipulation of offspring’s partners (Apostolou, 2013).

Not only parents but also the offspring can employ various behaviors to assert their interests. A study by Apostolou (2015) identified hypothetical counteractions (in the article called “countertactics”) used by the offspring to influence their parent’s mate choice decisions. They pertain to two hypothetical scenarios, where the offspring tries to manipulate the parents (1) into calling off a forced marriage and (2) into accepting a relationship the offspring desires. In the first hypothetical scenario, the study identified the following counteractions: Reason (e.g., the offspring explains why he/she does not like this particular person), Put yourselves in my shoes (e.g., offspring asks parents how they would feel if they were in the offspring’s shoes), Regression (e.g., the offspring cries to make the parents change their mind), the Silent treatment (e.g., the offspring stops speaking with the parents), and Threat (e.g., the offspring threatens the parents with secretly marrying another person) were categorized by the prior study of children’s influence on their parents within everyday interactions (Cowan et al., 1984). Further identified counteractions include I am committed elsewhere (e.g., the offspring tells parents he/she had already secretly married another person) and Suicide (e.g., the offspring attempts suicide). Counteractions used by the offspring within the second scenario were (as identified by Cowan et al., 1984) Asking for trust (e.g., the offspring tells parents he/she is an adult and capable of making own choices), Standing one’s ground (e.g., the offspring tells parents he/she will disobey them and continue with the relationship), Cajoling (e.g., the offspring tells parents the desired partner is very much like them which is why the offspring wants to stay with him/her), and, similar to counteractions in the previous scenario, Regression (e.g., the offspring cries to make the parents change their minds) and the Silent treatment (e.g., the offspring stops talking to the parents). Further identified strategies deployed by the offspring to influence parental behavior were Commitment (e.g., the offspring secretly marries the desired partner) and My mate is great! (e.g., the offspring tells parents that he/she cannot live without the desired partner).

In some cases, parental actions might conflict with the aims of the offspring’s partners who wish to continue in their relationship with the offspring. Apostolou (2017) therefore identified counteractions of the offspring’s partners within two hypothetical scenarios. In these cases, though, the content of these categories largely overlapped with particular behaviors: for instance, Talk to them to change their minds! (the partner tries to demonstrate to parents that he/she is the ideal partner for the offspring), I have strong feelings for your child (the partner demonstrates how serious he/she is about relationship with the offspring) both aim at maintaining a relationship while I do not open up to them (avoidance of opening up to partner’s parents), the Silent treatment (the offspring’s partner does not talk to the parents), and Avoidance (avoidance or ignoring of partner’s parents) are intended to keep the parents at a distance.

Earlier studies (Butkovic & Bratko, 2007; Cowan & Avants, 1988) that used a family design of children’ manipulation (defined here as ways in which individuals intentionally or purposefully influence or exploit others; Buss, 1987) aimed at parents in everyday contexts found a low agreement between self-reports and observer reports. This suggests that the perception of these actions significantly differs depending on the viewpoint, that is, the perspective of the manipulator vs the person who is manipulated. Importantly, quantitative studies (Apostolou, 2015, 2017; Buss, 1992; Cowan et al., 1984) have based their categorization solely on the offspring’s reports without considering the parent’s perspective, although evidence clearly shows that it makes a difference whether one looks at intergenerational relationships from the perspective of the parent or the offspring (Aquilino, 1999; Kim et al., 2014; Shapiro, 2004; Steinbach et al., 2019). Similarly, qualitative studies analyzed parental interventions aimed at the offspring based on single perspective, either that of the offspring (Bates, 1942) or that of the parents or other family members (Ikels, 1985; Sussman, 1953). Most studies on parental actions and the offspring’s and their partner’s counteractions are based on reports from young adults, mainly students (Apostolou, 2013, 2015, 2017; Cowan & Avants, 1988; Cowan et al., 1984), or on reports coming from the parents of prepubertal children who have little or no romantic experience (Apostolou, 2013). Importantly, though, evidence suggests that there exists a discrepancy between behavior in real life and in hypothetical scenarios (Baumeister et al., 2007; Camerer & Mobbs, 2017). One may thus wonder to what extent findings based on hypothetical scenarios reflect real experiences of parents and their offspring. Assessment of behavioral sets based on actual experiences from the perspective of both the adult offspring and their parents should thus contribute to a more realistic understanding of the processes involved in parental interventions.

Finally, previous studies mapped (prior to factor analysis) the actions or counteractions qualitatively through questionnaires (Apostolou, 2015), interviews (Apostolou, 2013), or both of these methods (Apostolou, 2017). This led to further limitations in the analytical part of the studies, for instance, because similar or vague actions were dropped or answers that contained multiple actions were excluded (Apostolou, 2017). In contrast, qualitative research with clearly stated steps of the theoretical analysis (Braun & Clarke, 2006) has the advantage of greater trustworthiness (Nowell et al., 2017) and the potential to generate unanticipated insights (Braun & Clarke, 2006).

The main aim of this study was exploratory: we want to describe sets of actions and counteractions from the perspective of both the offspring and the parents via semi-structured interviews of parent–offspring dyads. This method should afford us access to the behavioral patterns and impressions reflected in self-reports and observer reports in detail. In particular, we focus on actions used by parents to affect their offspring and the offspring’s partner and on the counteractions employed by the offspring to affect their parents. Unlike previous research which worked with hypothetical scenarios, our study investigates actual relationships and distinguishes between current and previous relationship contexts.

## Method

### Participants

A total of 40 participants took part in our study: 20 offspring (10 daughters and 10 sons) and one of their biological parents (10 mothers and 10 fathers). These participants formed five son–mother, five son–father, five daughter–mother, and five daughter–father dyads, because previous studies reported sex-dependent differences in parent–offspring dyad reports (Kim et al., 2014; Shapiro, 2004; Steinbach et al., 2019). Criteria for offspring’s participation were Czech nationality, age 18–45 years, being heterosexually identified in a committed long-term relationship (minimal relationship length six months with at least three months of cohabitation, perceived by participants as having a perspective for the future), growing up with the parent in the same household until at least 12 years of age (Štěrbová et al., 2019), and being in regular mutual contact (e.g., at family gatherings or by phone). Of the 20 offspring, three female participants had children (*M* = 6.75 years; SD = 3.96), two male participants had children (*M* = 6.94 years; SD = 4.81), and the partner of one of the male participants was pregnant. Participants reported the following relationship types: dating (*N* = 12), engaged (*N* = 5), and married (*N* = 3) dyads. Participants’ age, length of the relationship, and of cohabitation are reported in Table 1.

**Table 1:** Sample demographics

	Age (years)			Relationship length (years)		Length of partners’ cohabitation (years)	
	Range	M	SD	M	SD	M	SD
Daughters	23–40	30.3	5.46	8.08	5.65	5.9	5.12
Sons	25–44	32.5	5.82	4.85	5.75	3.86	5.96
Mothers	45–61	53.8	6.18				
Fathers	51–71	62.3	7.13				

**Fig. 1:**
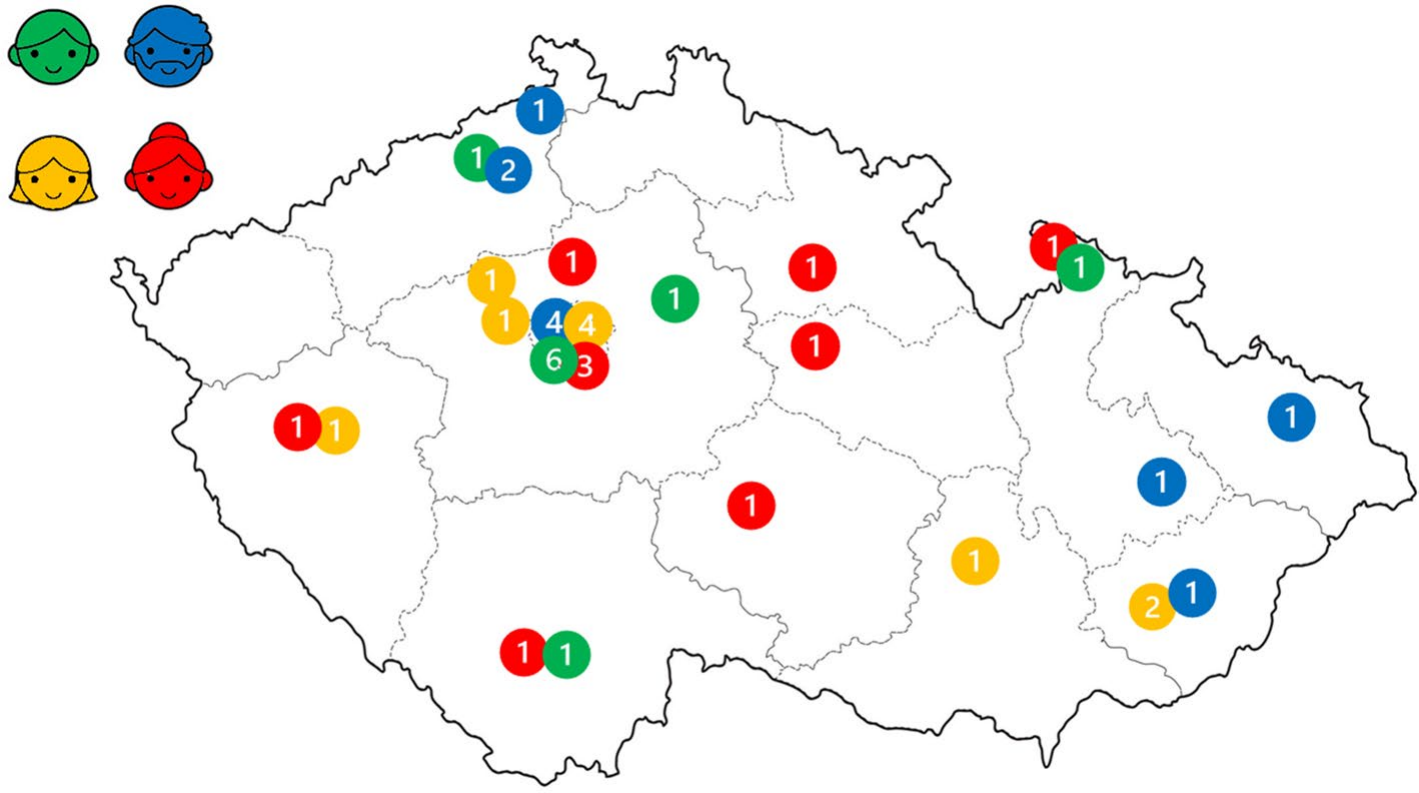
Participants’ current place of residence. Mothers are represented by red, fathers by blue, sons by green, and daughters by yellow color. Each number represents participants living in that area of the Czech Republic (Color figure online)

The sample size of 40 participants was established before the recruitment process. A similar sample size was established in a prior study by Apostolou (2013). Additionally, our aim was also to achieve sex-balanced parent–offspring dyads (see Procedure section). No further data collection was necessary because the data analysis indicated a theoretical saturation of the themes (Braun & Clarke, 2021). All participants came from the ethnically homogeneous Czech Republic. Their current location of residence is visualized in Fig. 1. Czech society is regarded as individualistic, with a score of 58 out of 100 points on the individualism–collectivism scale (Hofstede, 2010). Czech society is also one of the most secular ones in Europe with less conservative views of family and marriage (Hamplová, 2000).

### Measures

Each interview consisted of four parts (the same for offspring and their parents). The first part focused on how parents perceive the offspring’s partner, what they do and do not like about him or her, and whether the parent and the offspring discuss the offspring’s relationship. The second part investigated whether and how parents reacted at the beginning of their offspring’s current relationship, thus including, for instance, the parents’ impressions of the offspring’s partner or whether the parents tried to interfere in the relationship. The third part mapped whether and how the parents react to the offspring’s current relationship, for instance, whether they give feedback about the relationship to the offspring. Finally, we mapped how parents reacted to their offspring’s previous relationships, that is, their attitudes to the offspring’s ex-partners and whether (and why) the offspring did not start or end the relationship because of the parent. (For the full list of questions, see Supplementary material.) The interview structure was thus fixed but the number of questions varied depending on the number of the offspring’s previous experiences with romantic relationships or additional probing questions concerning, for instance, the behavior, attitudes, and impressions of the offspring or the offspring’s partner.

In June 2020, we conducted a pilot study with four dyads (son–mother, son–father, daughter–mother, and daughter–father) to assess whether participants perceive the interview questions as clear and comprehensible. Based on participants’ responses, we elaborated the questions about intervention actions. The first author, experienced in interview techniques, conducted all the interviews, including pilot interviews.

### Procedure

Participants were recruited online via social media (Facebook research groups, Instagram), leaflets distributed at the Charles University, Faculty of Science, via an email list of participants of previous studies (Štěrbová et al., 2019), and by snowball sampling. Respondents completed an online contact form (built on a Qualtrics platform) with information about the study followed by an informed consent form and a sociodemographic questionnaire. Maximum variation sampling (Suri, 2011) was employed regarding participants’ age, education level, net monthly income, place of residence, relationship status, relationship length, length of partners’ cohabitation, and the number of children, because these variables are likely to influence the choice and use of actions related to mate choice (Apostolou, 2013; Apostolou & Papageorgi, 2014). Individuals who met the recruitment criteria (see the Participants section) were contacted via email to schedule an interview. Parents and offspring were recruited separately to avoid the risk of undue mutual influence/coercion.

Semi-structured interviews were conducted in July–October 2020 with each participant separately and in person (except for two online interviews due to Covid-19 restrictions). At the beginning, all participants expressed their willingness to be interviewed by signing an informed consent form. Interviews ranged in length from 26 to 68 min (*M* = 47.32 min; SD = 11.37). Parent’s sociodemographic characteristics were gathered in the first part of the interview with each parent. Content of the second part of the interview was the same for the parents and the offspring, but the questions were asked from their own perspectives. Participants were reimbursed with 200 CZK (app. 10 USD). The study was approved by the Institutional Review Board of the Charles University, Faculty of Science (approval number 2020/05).

### Data Analysis

Transcripts from the interviews were transferred to qualitative data analysis software Atlas.ti 9.0. The analysis was guided by the method of theoretical thematic analysis, which enables data coding with no pre-existing coding frame or preconceptions. Rather, this approach is driven by the researcher’s theoretical or analytic interest. In line with the semantic (descriptive) approach, themes are generated from the data and strongly linked to codes (Braun & Clarke, 2006). We used five phases for thematic analysis coding (Braun & Clarke, 2006) as recommended by Nowell and colleagues (2017) to strengthen trustworthiness. First, we familiarized ourselves with the data by listening to interview recordings, by repeated independent reading of interview transcripts by two coders (the first and the last author), and by taking notes about ideas for coding to be used in the subsequent phases. In the second phase, the two coders together generated initial codes from the first four transcripts. This was followed by a systematic discussion and code comparison aimed at giving full and equal attention to each and every data item from the dataset (Braun & Clarke, 2006) and at a reduction of interchangeability and redundancy (Attride-Stirling, 2001). After coders reached a consensus on the codebook, the first coder (the first author) coded all the remaining transcripts while in continuous discussions about the process with the second coder. This was followed by discussions with a third researcher (the second author); in other words, by researcher triangulation to verify preliminary findings (Kalpokaite & Radivojevic, 2020; Nowell et al., 2017).

What was coded was either the reported behavior (e.g., “I tried to calm her down”) or its direct labeling, that is, the respondent’s impressions/feelings (e.g., “I provide my offspring with emotional support”). We coded only those behaviors, impressions, and feelings of the parent–offspring dyad which were related to the offspring’s current or previous relationships. During the coding process, we also identified parental actions targeting both the offspring and the partner, that is, the couple as such. Importantly, besides the offspring’s counteractions we identified in respondent’s spontaneous responses also counteractions undertaken by the offspring’s partner, which were directed at the offspring’s parent. Moreover, we considered it important to differentiate between independently pursued behavior (actions) of the offspring or the offspring’s partner as opposed to reactions to parental interference (counteractions).

In the third phase of the coding process, the first coder started to create themes from the initial codes. Each code was ascribed to one theme based on content similarity. The fourth phase, which consisted in reviewing the themes, was accompanied by repeated discussions. Some themes were renamed, others with similar meanings merged, and yet others were split by the first author. During re-reading of the data, the first coder created some new codes that were incorporated into the themes, while other codes were merged because of high similarity of content. Furthermore, we created new code groups indicating the actor (who performed the theme: the parent, the offspring, the offspring’s partner) and the recipient (at whom it is directed: the parent, the offspring, the offspring’s partner, or the couple). In the fifth phase, we defined and labeled the final set of themes based on the consensus of the entire team (the first, second, and the last author) achieved by repeated reading of the data and checking of the coding process. To maintain links between the themes and the codes, we constantly compared them (Pope, 2000) during the process of data analysis.

To identify the sets of actions taken by the offspring, offspring’s partners, and the parents as well as the sets of actions/counteractions taken by the offspring and the offspring’s partner, we have manually selected themes used by particular actors toward specific recipients. In some cases, respondents spontaneously reported certain behaviors as being used by both of their parents, so we coded these actions under parental actions ascribed to the parent from the interviewed dyad. On the other hand, reported actions or counteractions concerning the other parent, i.e., the parent who was not interviewed, were not included in the coding process and neither were other behaviors that had no direct bearing on the offspring’s relationship. Further, the actor–recipient sets of actions and counteractions were accompanied by the number of reports by the mothers, fathers, daughters, and sons.

For easier orientation in the Results and Discussion sections, we categorized the parents’ actions based on their impact on the offspring’s relationship (identified either by the respondent or the first author, depending on the context). Some parental actions were neither clearly supportive nor clearly disruptive, or their intended impact on the couple was unclear or unreported. We classified such actions as ambivalent. For example, Giving advice might be either supportive or disruptive. Similarly, when it comes to Talking about the offspring’s partner it depends on the context and the intention; as such, the action is thus ambivalent.

## Results

### Sets of Actions Reported by the Parents and the Offspring

Thematic analysis identified 32 parental actions aimed at the offspring (see Table 2), their partners (see Table 3), or the couple as a whole (see Table 4). Table 5 shows the categories of parental actions classified as ambivalent, supportive, or disruptive based on their relation to the offspring’s relationship. Furthermore, we identified 13 counteractions undertaken in response to parental interventions and five independently pursued actions, that is, actions taken regardless of parental behavior by the offspring (see Table 6) and/or their partners (for further details, see Table 7) aimed at the offspring’s parents.

**Table 2:** The set of parents’ actions aimed at the offspring

Actions reported by the offspring	Daughters	Sons	Total	Actions reported by the parents	Mothers	Fathers	Total
Talking about the offspring’s partner	10	9	19 (95%)	Talking about the offspring’s partner	8	8	16 (80%)
Giving feedback to the offspring	10	7	17 (85%)	Giving feedback to the offspring	10	5	15 (75%)
Not giving any feedbacks or advice	5	10	15 (75%)	Not giving any feedbacks or advice	5	10	15 (75%)
Respecting the offspring’s attitude	7	6	13 (65%)	Respecting the offspring’s attitude	6	6	12 (60%)
Questioning/stalking	8	3	11 (55%)	Giving advice	8	1	9 (45%)
Dissenting attitude/criticism of the offspring’s partner	9	2	11 (55%)	Dissenting attitude/criticism of the offspring’s partner	5	3	8 (40%)
Giving advice	7	2	9 (45%)	Praising the offspring’s partner	7	0	7 (35%)
Emotional support	5	3	8 (40%)	Questioning/stalking	5	1	6 (30%)
Instigation of questioning of the relationship	5	2	7 (35%)	Instigation of questioning of the relationship	4	1	5 (25%)
Expressing fear for the offspring	4	2	6 (30%)	Expressing fear for the offspring	4	0	4 (20%)
Defending the offspring/offspring’s partner	3	3	6 (30%)	Controlling the offspring	2	2	4 (20%)
Emotional pressure/blackmail	4	1	5 (25%)	Emotional support	3	0	3 (15%)
Giving feedback to the offspring on his/her ex-partner ex post	2	3	5 (25%)	Defending the offspring/offspring’s partner	3	0	3 (15%)
Practical/material support	4	1	5 (25%)	Practical/material support	1	2	3 (15%)
Disrespect/interfering with/violation of the autonomy of the partner and the offspring	4	0	4 (20%)	Spending time together	2	0	2 (10%)
Praising the offspring’s partner	2	2	4 (20%)	Emotional pressure/blackmail	2	0	2 (10%)
Comparing offspring’s current partner with his/her ex-partner	2	1	3 (15%)	Disagreement with offspring's behavior	1	1	2 (10%)
Controlling the offspring	2	0	2 (10%)	Demanding help, care, attention	1	1	2 (10%)
Disagreement with the offspring’s behavior	1	1	2 (10%)	Comparing offspring’s current partner with his/her ex-partner	0	2	2 (10%)
Spending time together	1	0	1 (5%)	Giving feedback to the offspring on his/her ex-partner ex post	0	1	1 (5%)
Hypocritical behavior	1	0	1 (5%)	Informing the other parent about the offspring’s relationship	1	0	1 (5%)
Friendly behavior toward the offspring’s partner	1	0	1 (5%)				
Demanding help, care, attention	1	0	1 (5%)				
Informing the other parent about the offspring’s relationship	1	0	1 (5%)				
Making fun of the offspring/offspring’s partner	0	1	1 (5%)

**Table 3:** The set of parents’ actions aimed at the offspring’s partner

Actions reported by the offspring	Daughters	Sons	Total	Actions reported by the parents	Mothers	Fathers	Total
Practical/material support	6	6	12 (60%)	Practical/material support	5	2	7 (35%)
Friendly behavior toward the offspring’s partner	7	4	11 (55%)	Standoffish behavior	2	4	6 (30%)
Questioning/stalking	6	0	6 (30%)	Questioning/stalking	3	2	5 (25%)
Standoffish behavior	4	2	6 (30%)	Praising the offspring’s partner	3	1	4 (20%)
Hypocritical behavior	2	1	3 (15%)	Defending the offspring/offspring’s partner	4	0	4 (20%)
Giving advice	2	1	3 (15%)	Friendly behavior toward the offspring’s partner	2	2	4 (20%)
Praising the offspring’s partner	2	1	3 (15%)	Maintaining contact with the ex-partner of the offspring	2	1	3 (15%)
Dissenting attitude/criticism of the offspring’s partner	2	1	3 (15%)	Dissenting attitude/criticism of the offspring’s partner	2	1	3 (15%)
Spending time together	1	1	2 (10%)	Making fun of the offspring/offspring’s partner	2	1	3 (15%)
Emotional pressure/blackmail	2	0	2 (10%)	Giving advice	2	1	3 (15%)
Demanding help, care, attention	2	0	2 (10%)	Spending time together	2	0	2 (10%)
Ganging up with the offspring’s partner against the offspring	1	1	2 (10%)	No financial/material support	0	2	2 (10%)
Not giving any feedback or advice	1	0	1 (5%)	Demanding help, care, attention	1	1	2 (10%)
No financial/material support	0	1	1 (5%)	Inducing a situation where the characteristics of the offspring’s partner are uncovered	1	0	1 (5%)
Disrespect/interfering with/violation of the autonomy of the partner and the offspring	0	1	1 (5%)	Expressing fear for the offspring	1	0	1 (5%)
Making fun of the offspring/offspring’s partner	0	1	1 (5%)	Emotional pressure/blackmail	1	0	1 (5%)
				Not giving any feedback or advice	0	1	1 (5%)
				Disrespect/interfering with/violation of the autonomy of the partner and the offspring	0	1	1 (5%)

**Table 4:** The set of parent’s actions aimed at the couple

Actions reported by the offspring	Daughters	Sons	Total	Actions reported by the parents	Mothers	Fathers	Total
Spending time together	7	8	15 (75%)	Spending time together	7	8	15 (75%)
Practical/material support	3	2	5 (25%)	Practical/material support	4	4	8 (40%)
Giving up on interventions/engagement	2	0	2 (10%)	Giving up on interventions/engagement	3	2	5 (25%)
Offering help	1	1	2 (10%)	Emotional support	2	1	3 (15%)
Disrespect/interfering with/violation of the autonomy of the partner and the offspring	1	1	2 (10%)	No financial/material support	1	2	3 (15%)
Giving advice	1	1	2 (10%)	Offering help	2	0	2 (10%)
Emotional pressure/blackmail	1	0	1 (5%)	Giving advice	2	0	2 (10%)
Emotional support	1	0	1 (5%)	Emotional pressure/blackmail	0	1	1 (5%)
Controlling the offspring	0	1	1 (5%)	Disrespect/interfering with/violation of the autonomy of the partner and the offspring	1	0	1 (5%)
				Talking about the offspring’s partner	0	1	1 (5%)
				Praising the offspring’s partner	1	0	1 (5%)

**Table 5:** Categorization of parents’ actions

Ambivalent	Supportive	Disruptive
Giving advice	Comparing offspring’s current partner with his/her ex-partner	Controlling the offspring
Giving feedback to the offspring	Defending the offspring/offspring’s partner	Demanding help, care, attention
Giving feedback to the offspring on his/her ex-partner ex post	Emotional support	Disagreement with the offspring’s behavior
Informing the other parent about the offspring’s relationship	Friendly behavior toward the offspring’s partner	Disrespect/interfering with/violation of the autonomy of the partner and the offspring
No financial/material support	Offering help	Dissenting attitude/criticism of the offspring’s partner
Not giving any feedback or advice	Practical/material support	Emotional pressure/blackmail
Talking about the offspring’s partner	Praising the offspring’s partner	Expressing fear for the offspring
	Giving up on interventions/engagement	Ganging up with the offspring’s partner against the offspring
	Respecting the offspring’s attitude	Hypocritical behavior
	Spending time together	Inducing a situation where characteristics of the offspring’s partner are uncovered
		Instigation of questioning of the relationship
		Maintaining contact with the ex-partner of the offspring
		Making fun of the offspring/offspring’s partner
		Questioning/stalking
		Standoffish behavior

**Table 6:** The set of offspring’s counteractions and actions aimed at the parents

Actions/counteractions reported by the offspring	Daughters	Sons	Total	Actions/counteractions reported by the parents	Mothers	Fathers	Total
Confiding/talking about the partner	10	10	20 (100%)	Confiding/talking about the partner	10	8	18 (90%)
Spending time together	6	5	11 (55%)	Not confiding/not talking about the partner	4	8	12 (60%)
Opposition/disobeying the parent^C^	8	2	10 (50%)	Spending time together	4	7	11 (55%)
Not confiding/not talking about the partner	4	5	9 (45%)	Opposition/disobeying the parent^C^	5	3	8 (40%)
Compliance with parents’ advice/rules^C^	4	0	4 (20%)	Compliance with parents’ advice/rules^C^	4	2	6 (30%)
Lying to the parent/withholding information from the parent^C^	3	0	3 (15%)	Explaining, advocating, justifying^C^	3	1	4 (20%)
Explaining, advocating, justifying^C^	2	1	3 (15%)	Ignoring the parent^C^	1	2	3 (15%)
Ignoring the parent^C^	1	2	3 (15%)	Asking the parent for practical/material help	0	1	1 (5%)
Asking the parent for practical/material help	2	0	2 (10%)	Friendly/helpful behavior toward the parent^C^	0	1	1 (5%)
Reducing the frequency of visits/separation from the family^C^	1	0	1 (5%)	Reducing the frequency of visits/separation from the family^C^	1	0	1 (5%)
Giving up on explaining/resignation regarding parent's engagement^C^	1	0	1 (5%)	Lying to the parent/withholding information from the parent^C^	1	0	1 (5%)
Accepting help/invitation^C^	1	0	1 (5%)	Accepting help/invitation^C^	1	0	1 (5%)
Praising the partner	1	0	1 (5%)	Praising the partner	1	0	1 (5%)

“C” means a counteraction, i.e., a defensive reaction to parental interferences, as contrasted with “action”, an independently pursued behavior of the offspring or their partners when asserting their interests

**Table 7:** A set of actions and counteractions used by the offspring’s partner in relation to the offspring’s parents

Actions/counteractions reported by the offspring	Daughters	Sons	Total	Actions/counteractions reported by the parents	Mothers	Fathers	Total
Spending time together	6	6	12 (60%)	Spending time together	4	7	11 (55%)
Opposition/disobeying the parent^C^	3	4	7 (35%)	Friendly/helpful behavior toward the parent^C^	4	2	6 (30%)
Friendly/helpful behavior toward the parent^C^	4	2	6 (30%)	Opposition/disobeying the parent^C^	4	2	6 (30%)
Explaining, advocating, justifying^C^	3	0	3 (30%)	Confiding/talking about the partner	1	1	2 (10%)
Criticizing the offspring’s parent to the offspring	2	1	3 (30%)	Explaining, advocating, justifying^C^	2	0	2 (10%)
Compliance with parents’ advice/rules^C^	2	0	2 (10%)	Reducing the frequency of visits/separation from the family^C^	0	2	2 (10%)
Reducing the frequency of visits/separation from the family^C^	0	2	2 (10%)	Limiting the offspring’s free time^C^	1	1	2 (10%)
Ganging up with the offspring’s parent against the offspring^C^	1	1	2 (10%)	Asking the parent for practical/material help	0	1	1 (5%)
Not confiding/not talking about the partner	0	1	1 (5%)	Compliance with parents’ advice/rules^C^	0	1	1 (5%)
Giving up on explaining/resignation regarding parent's engagement^C^	1	0	1 (5%)	Telling the offspring about parental interventions	1	0	1 (5%)
Telling the offspring about parental interventions	1	0	1 (5%)	Acceptance of help/invitation^C^	1	0	1 (5%)
Praising the offspring	1	0	1 (5%)	Ignoring the parent^C^	1	0	1 (5%)
Limiting the offspring’s free time^C^	0	1	1 (5%)				

“C” means a counteraction, i.e., a defensive reaction to parental interferences, as contrasted with “action”, an independently pursued behavior of the offspring or their partners when asserting their interests

Most actions or counteractions identified by the thematic analysis were reported in relation to both the past and the current relationships; for this reason, the two potential categories (past/present relationship) were merged into one. Overall, daughters and mothers reported more actions or counteractions than fathers and sons did and that with respect to all of the actor–recipient contexts. The use of certain actions and counteractions to some degree depended on the actor and the recipient and this was reported from both the parents’ and the offspring’s perspectives.

### Actions Employed by Parents

Table 2 shows parental actions aimed at the offspring from both the parent’s and the offspring’s perspectives. Interestingly, compared to parents, the offspring reported four additional parental actions: Disrespect/interfering with/violation of autonomy of the partner and the offspring, Hypocritical behavior, Friendly behavior toward the offspring's partner, and Making fun of the offspring/offspring’s partner. To illustrate the last-mentioned action, we add a quotation from a son (D5) giving an example of his father’s jokes and comments:

So, we’re sitting down for a family lunch (chuckles) and my dad starts wondering whether [the son’s girlfriend] has already finished her school and then he mentions it, he makes fun of [her], because he’s a technician and for him, it’s the practical side of things that’s important, and he hints (…) that it’ll be difficult to get a job and things like that.

Table 3 shows actions deployed by parents toward the offspring’s partners. Compared to actions aimed at the offspring, we uniquely identified Standoffish behavior, No financial/material support reported by both the parents and the offspring, and Ganging up with the offspring’s partner against the offspring that was reported only by the offspring also as Hypocritical behavior. Parents reported four actions not reported by the offspring: Defending the offspring/offspring’s partner, Expressing fear for the offspring, and two actions found only in relation to the offspring’s previous relationship, namely Maintaining contact with the ex-partner of the offspring and Inducing a situation where characteristics of the offspring’s partner are uncovered.

Table 4 shows actions applied by parents toward the couple. Compared to actions toward the offspring and offspring’s partner, we uniquely identified Giving up on interventions/engagement and Offering help reported by both parents and offspring. One additional action was not reported by parents in relation to the offspring’s prior relationship, namely Controlling the offspring. Parental control was reported in our sample in both the parents and the offspring. A daughter (D3), for instance, openly spoke about her mother’s control practices:

For example, my mom was forbidding me to go to his [ex-boyfriend’s] house, but then I did it in secret and like that, so then she was glad I actually told her and she knew where I was. But at first, the first half a year when we started dating, I was just forbidden to go anywhere with him, like in a car and such. That they just wanted us to at least stay in town and didn’t want me to go to his house.

Three parental actions were not reported by the offspring (No financial or material support, Talking about the offspring’s partner, and Praising the offspring’s partner).

### Counteractions and Actions Applied by Offspring

Table 6 displays a set of offspring’s counteractions and actions aimed at the parent. Overall, we found more reports of counteractions as defensive reactions to parental behavior (for example, Opposition/disobeying the parent, Explaining, advocating, justifying, Compliance with parents’ advice/rules, Reduction of frequency of visits/separation from the family) than offspring’s self-initiated actions. Those we did find referred more to neutral, non-conflict behavior, such as Confiding/talking about the partner, Spending time together, or *Praising the partner*. Both groups, i.e., both the parents and the offspring, reported a similar frequency of most counteractions and actions taken by the offspring. Unlike the offspring, parents reported Friendly/helpful behavior toward the parent, while the offspring reported Giving up on explaining/resignation regarding parent’s engagement without any counterpart on the parent’s part.

### Counteractions and Actions Undertaken by Offspring’s Partner

In the case of actions and counteractions used by the offspring’s partner toward the parent, similarly, more frequently reported were counteractions rather than independent actions. Parents reported Confiding/talking about the partner, Asking the parent for practical/material help, Acceptance of help/invitation, and Ignoring the parent without any counterpart on the part of the offspring. On the other hand, Criticizing the offspring’s parent to the offspring, Ganging up with the offspring’s parent against the offspring, Not confiding/not talking about the partner, Giving up on explaining/resignation regarding parent's engagement, and Praising the offspring were actions which did not have a counterpart on the parent’s side (see Table 7 for further details).

### Behavioral Patterns Regarding Current vs. Previous Relationship

Since the interviews focused primarily on behavioral patterns related to the context of the offspring’s current relationship, most actions and counteractions we identified related to it. Nevertheless, five parental actions were reported only in relation to the current and not the previous relationship of the offspring: Offering help, Disagreement with the offspring’s behavior, Ganging up with the offspring’s partner against the offspring, Praising the offspring’s partner, and Informing the other parent about the offspring’s relationship. Further, we identified three parental actions reported only in relation to the offspring’s previous relationship: Inducing a situation where characteristics of the offspring’s partner are uncovered, Giving feedback to the offspring on his/her ex-partner ex post, and Maintaining contact with the ex-partner of the offspring. The following quotation from a mother (D7) illustrates being in touch with her son’s ex-girlfriend despite their breakup: “I think she [the son’s ex-girlfriend] was quite fond of me even after the breakup; actually, we saw each other from time to time, and she would look me up.”

Regarding counteractions of the offspring or his or her partner, Telling the offspring about parental interventions, Accepting help/invitation, Ganging up with the offspring’s parent against the offspring, Criticizing the offspring’s parent to the offspring, and Ignoring the parent were reported in relation to the current relationship. The only action reported in the context of the current but not the previous relationship was Praising the partner. One counteraction, namely Limiting the offspring’s free time, was reported only in relation to the offspring’s previous relationship. The last-named counteraction is exemplified by a quotation from interview with a father (D19): “She [the son’s ex-girlfriend] was… she was basically preventing him [the son] from meeting people, she wanted him all to herself… so we would not see him even for like half a year.”

## Discussion

Based on semi-structured interviews with 20 parents and 20 offspring, we have identified parental actions aimed at their offspring and the offspring’s partner. Importantly, we also found that parental actions directed at both the offspring and their partners jointly, that is, at the couple, did not tend to be as disruptive or ambivalent as actions applied separately to the offspring or the offspring’s partner. Instead, they tended to be mostly supportive. The form of parental interference thus differed depending on the recipient. Further, we found that not only the offspring but also their partners react to parental interventions by counteractions. Interestingly, the thematic analysis revealed that both the offspring and their partners also pursue their own aims, regardless of parental interference, by taking independent actions aimed at the offspring’s parents. In the current study, we expanded the list of previously reported parental actions (Apostolou, 2013; Bates, 1942; Buss, 1992; Ikels, 1985; Sussman, 1953) and the list of counteractions taken by the offspring (Apostolou, 2015) and offspring’s partners (Apostolou, 2017), and we identified parental actions directed jointly at the offspring and their partners, i.e., at the couple. Our sets of actions can thus be employed in future studies testing parental influence of their offspring’s mate choice.

### Parental Actions Aimed at the Offspring

The set of parental actions aimed at the offspring tended to be the largest according to reports of both the parents and the offspring. Importantly, we further expanded the set of parental actions reported by previous research (Apostolou, 2013) to include actions which are ambivalent (for example, Talking about the offspring’s partner, Giving feedback to the offspring, Not giving feedback or advice) or supportive ones (for example, Respecting the offspring’s attitude, Emotional support, Practical/material support, Defending the offspring/offspring’s partner, Spending time together). Our findings thus support previous reports of high parental investments in financial, practical, and emotional support, including listening and talking about events from the daily life of the adult offspring (e.g., Fingerman et al., 2012; Kim et al., 2014). Interestingly, the parents in our sample provided emotional support to the offspring and to the couple, but not to the offspring’s partner. One parent and one offspring in our sample explicitly reported that parental support goes exclusively to the offspring, regardless of the partner:

It’s like we do support our offspring in what he’s doing, right, so if he said like, “I wanna break up with her [his girlfriend], I don’t want her,” we’d support him. If he says “I wanna stay with her," we’ll support him as well… (Father, D5)

Son (D8) similarly reported:

Sure, but this support [emotional support by parents] doesn’t affect my relationship, I think. It would be there even if I was alone or had any other girlfriend, so we’d probably be always welcomed in their [the parents’] house.

In our study, most parental actions aimed at the offspring partly overlap with prior findings (Apostolou, 2013; Bates, 1942; Buss, 1992; Ikels, 1985; Sussman, 1953). For example, Coercion and Guilt trip are similar in content to our Emotional pressure/blackmail, Chaperoning is similar to what we call Controlling the offspring (Apostolou, 2013), while Hardball corresponds in our sets to various categories of actions including Dissenting attitude/criticism of the offspring’s partner, Instigation of questioning of the relationship, Disagreement with the offspring’s behavior (Apostolou, 2013; Buss, 1992), and Advice/Reasoning and Whom one should marry, which corresponds to our Giving advice (Apostolou, 2013; Bates, 1942; Ikels, 1985; Sussman, 1953). Interestingly, we did not identify any Use of relatives and friends, represented, for instance, by asking a relative or friend to try and influence offspring to do something (Apostolou, 2013). What we did find was a similar use of friends in the Questioning/stalking behavior, represented, for instance, by checking offspring’s answers to their friends, or Informing the other parent about the offspring’s relationship, which might motivate the other parent’s further action. It is possible that due to the predominantly nuclear family structure in Czech society (Czech Statistical Office, 2020), parents might use for indirect interventions friends rather than other family members whose involvement has been reported in countries with more extended family structure, such as Cyprus (Apostolou, 2013).

### Parental Actions Aimed at the Offspring’s Partner

In the case of parental actions aimed at the offspring’s partner, the most applied parental behavior we found was disruptive, e.g., Questioning/stalking or Standoffish behavior followed by supportive actions, such as Practical/material support. Because offspring’s mate choice affects the whole family (Faulkner & Schaller, 2007), parents probe the suitability of their offspring’s partner to prevent the potential costs of a long period of parental investment, secure heritability of resources, and to maintain the functioning of extensive networks of kinship and reciprocity (see Apostolou, 2007).

Three out of four parental actions aimed at the offspring’s partner are similar in content to those reported by prior studies (Apostolou, 2013; Buss, 1992): Hardball (here represented by Dissenting attitude/criticism of the offspring’s partner, Questioning/stalking, Making fun of the offspring/offspring’s partner), We are family (here named Practical/material support and Friendly behavior toward the offspring’s partner), and Dirty laundry (here called Questioning/stalking). Our study did not identify any instance of Lure, e.g., promises of financial support, house, and money when the offspring’s partner marries the offspring. Instead, we found No financial/material support of the partner. Interestingly, we identified an action similar to Lure in Praising the offspring’s partner in the sense of trying to match the offspring with another/future partner.

### Parental Actions Aimed at the Couple

Regarding parental actions aimed at the couple, our results show that parents use supportive actions, such as Giving up on interventions/engagement, and pursue their intentions more subtly, for instance, by Offering help, and these actions are aimed only at the couple as a whole. It seems that parents do not apply such varied disruptive actions when facing the offspring and their partner together in comparison with actions undertaken when parents deal with either member of the couple separately, such as Dissenting attitude/criticism of the offspring’s partner or Questioning/stalking. It seems therefore that parents anticipate that they would not achieve their aims if faced with defensive or dissenting behavior from both members of the couple.

In the case of offspring’s actions aimed at the parent, mostly identified were neutral, non-conflict behavior, for example, Confiding/talking about the partner, Spending time together, and Not confiding/not talking about the partner. Offspring’s counteractions were ranging from conformation, for example, Compliance with parents’ advice/rules, to demarcation, such as Reducing the frequency of visits/separation from the family or Ignoring the parent, but these were deployed least frequently. This contrasted with the frequency of offspring’s counteractions identified in extreme hypothetical scenarios, for instance, counteractions aimed at manipulating parents to call off a forced marriage as reported by Apostolou (2015).

The fact that the most frequent actions were those of an ambivalent or supportive nature coming from the parents and not extreme reactions of the offspring or their partners is in part due to the method we used. Semi-structured interviews enabled us to investigate actual experienced interactions of parent–offspring dyads in detail without the need for any direct questions regarding the influence or interference of the parents or others, i.e., question about how the relatives, or friends’ parents influenced the relationship of the offspring (e.g., Apostolou, 2013). Hypothetical scenarios (Apostolou, 2015, 2017) may fail to identify nuanced behavioral patterns in everyday situations (Seidman, 2006), which are a natural consequence of the fact that parents and their adult offspring tend to be in regular contact (Fingerman et al., 2012). In general, the mutual parent–offspring relationship tends to gradually improve as the offspring get older (Tighe et al., 2016). The likelihood of extreme behavioral patterns on the part of either the parents or the offspring thus seems to slowly decrease with the offspring’s age (Apostolou, 2007).

### Offspring’s Counteractions and Actions Aimed at the Parents

In our study, we found no instances of offspring’s extreme behavior, such as I am committed elsewhere (telling the parent that the offspring has already secretly married another person) or Suicide (a suicide attempt on the part of the offspring; Apostolou, 2015). But there could still be some specific counteractions which the offspring might deploy both in extreme and everyday situations in response to parental interference. Some of the offspring’s behavior we observed partly overlapped with actions observed in the hypothetical Unacceptable in-laws or Forced mate scenarios, ranging from Asking for trust (in our study Opposition/disobeying the parent and Explaining, advocating, justifying), My mate is great! (here named Praising the partner) all the way to the Silent treatment (which corresponds to our Ignoring the parent or Reducing the frequency of visits/separation from the family; Apostolou, 2015). The occurrence of harsh and coercive behavioral patterns might be explained by the closeness of relationship between parents and their offspring, which can lead to the parties acting and reacting with less censorship of own reactions. Both parties can thus expect a higher tolerance or more efficient enforcement of their own interests (Riesch et al., 2003). Moreover, because our study worked with parent–offspring dyads, we assume that those who have a problematic relationship (e.g., offspring being blackmailed by the parent) would not participate in this type of study.

### Offspring’s Partner’s Counteractions and Actions Aimed at the Parents

This study identified a set of actions and counteractions undertaken by the offspring’s partner, among which Spending time together with the parent was one of the most frequently observed in both the offspring’s partner and the offspring. Unlike the offspring, the offspring’s partner did not use Lying to the parent/withholding information from the parent, reacting instead with sociable behavior, such as Friendly/helpful behavior toward the parent and Ganging up with the offspring’s parent against the offspring. Interestingly, due to their awareness of lifetime stability of the parent–offspring relationship (Zarit & Eggebeen, 2002), the offspring’s partners may want to prevent possible conflicts and disruptions in family coalitions when reacting to or independently acting on their disagreements with the parents directly. As a result, they tend to choose indirect approaches, such as Limiting the offspring’s free time, Criticizing the offspring’s parent to the offspring, and Telling the offspring about parental interventions.

### Sex Differences in Reported Actions and Counteractions

In our sample, mothers reported more actions or counteractions aimed at themselves, the offspring, the offspring’s partners, or the couple than the fathers did. Still, the current study is qualitative which is why any quantitative findings should be interpreted with caution. Overall, women tend to pay more attention to relations of and among their kin then men do (Faulkner & Schaller, 2007). Mothers thus also tend to be more involved than fathers in the mate choice of their offspring (Apostolou, 2011a, 2013). Offspring’s poor mate choice is likely to be more costly to mothers compare to fathers because mothers lose their own ability to reproduce with an onset of menopause and tend to invest more in grandparenting (Laham et al., 2005; Michalski & Shackelford, 2005). The fathers in our sample spoke little about their parental interference, more often reporting—unlike the mothers—Not giving any feedback or advice and No financial/material support. Lower levels of paternal interference might be rooted in uncertainty over paternity (Apostolou, 2011a) or in the fathers’ tendency to impose their influence on the offspring’s mate choice directly, e.g., by marriage arrangements (Apostolou, 2010, 2012), rather than indirectly, which is what mothers tend to opt for (Apostolou, 2013).

In line with prior findings (Apostolou, 2015), daughters reported more actions and counteractions aimed at the parents, themselves, or their male counterparts than the sons did. More daughters than sons thus reported Opposition/disobeying the parent, while sons reported no Compliance with parents’ advice/rules and no Lying to parent/withholding information from the parent. Sons reported more frequently than daughters Not getting any feedback or advice from parents and less frequently or not at all disrespectful parental behavior toward themselves or their female counterparts (such as Questioning/stalking). Prior quantitative studies (e.g., Apostolou, 2011b; Kuhle et al., 2015) showed that parents try to influence their daughters’ choices more often than the son’s choices. These differences can be explained by the asymmetry of parental investment (Trivers, 1974), where the minimum obligatory investment into reproduction (and often aftercare) and is higher in daughters compared to sons.

### Discrepancies Between Parents and Offspring in Reported Behavior

Our study is unique in investigating the complementary perspectives of both the parents and the offspring. Earlier studies had shown significant levels of discrepancy between reports of the parents and the offspring. For example, parents have a tendency to report higher levels of closeness (Aquilino, 1999; Steinbach et al., 2019; Van Houdt et al., 2020) and lower levels of conflicts (Aquilino, 1999; Shapiro, 2004; Steinbach et al., 2019), less ambivalence toward their offspring (Willson et al., 2006), and less giving of advice (Kim et al., 2014) than their adult offspring report. Along similar lines, in our study, parents—in contrast to their offspring—did not report some disruptive behavioral patterns toward the offspring, such as Disrespect/interfering with/violation of the autonomy of the partner and the offspring or Making fun of the offspring/offspring’s partner. Also in our study, parents reported behaviors such as Defending the offspring/offspring’s partner, which was reported by none of the offspring. These discrepancies can be explained by the self-enhancement theory, which describes a tendency to positive bias in reporting one’s own actions (e.g., giving support) as opposed to actions of other persons (Krueger, 1998). Other discrepancies, that is, actions reported only by the offspring, include Hypocritical behavior toward the offspring and the partner, might be explained by each party simply interpreting the intentions of the other party differently (Butkovic & Bratko, 2007; Cowan & Avants, 1988; Kim et al., 2014) or even by lack of awareness of particular actions, especially when the behavior in question was intentionally confidential or secret. For instance, Maintaining contact with the ex-partner of the offspring and Inducing a situation where characteristics of the offspring’s partner are uncovered were reported only by parents in relation to the offspring’s previous relationships.

Similar patterns of discrepancy were found in reports about the offspring’s or their partners’ actions and counteractions aimed at the parent. Giving up on explaining/resignation regarding parent's engagement or Ganging up with the offspring’s parent against the offspring as reported by the offspring was not reflected by parents. Interestingly, Asking the parent for practical/material, Acceptance of help/invitation help, and Confiding/talking about the partner applied by offspring’s partner were reported by parents but not by the offspring. This discrepancy might be due to the parents’ self-enhancing tendency that took the form of demonstrating helpful intentions or due to the offspring’s avoidance of feelings of dependence. An earlier study by Kim and colleagues (2014) found that in general, adult offspring who are more dependent on their parents tend to report receiving less practical and financial help than their parents report providing.

### Current vs. Previous Relationship Context

It should be emphasized that this study identified more actions or counteractions related to the current as opposed to previous relationships of the offspring because the interview questions concerned mainly the current relationship. Moreover, retrospective reflection upon one’s own behavior or the behavior of others might lead to lower numbers of reported actions or behaviors due to lower recall (Bell & Bell, 2018). Importantly, actions and counteractions reported in relation to previous relationships are contextually determined by the offspring being younger and, in most cases, also more dependent on the parents (in many cases, they still lived in the same household or received financial support from them). These previous romantic relationships were presumably not as stable as the current ones and in many cases—reported either by the offspring or by the parent—the parents strongly disapproved of the partners. Parental actions reported solely in relation to the previous relationships were Inducing a situation where characteristics of the offspring’s partner are uncovered, Giving feedback to the offspring on his/her ex-partner ex post, and Maintaining contact with the ex-partner of the offspring.

### Limitations and Future Research Directions

Due to the time constraints of the method of semi-structured interviews (Seidman, 2006), most questions targeted interfering parental behavior. Therefore, the higher occurrence of counteractions by the offspring or by their partners compared to actions initiated by themselves might be biased, because participants were responding to questions about parental interference. Further, because we interviewed parent–offspring dyads, we recruited only dyads where both parties were willing to participate. It can be thus assumed that the parents and offspring in our sample had disproportionally good relationships and, as a consequence, some of the harsher or coercive behaviors may have been, in our sample, either absent and/or unreported.

Our sample was rather homogeneous. In heterogeneous samples consisted of individuals with various characteristics (e.g., significant age variance between the parent–offspring or between the offspring–partner relationship), this might elicit a specific use of parental actions or offspring’s or offspring’s partner’s counteractions (Apostolou, 2017). Future studies should consider the effect of various sociodemographic and cultural background (Apostolou & Papageorgi, 2014) on the use of particular actions/counteractions.

Interestingly, in our study we identified an indirect parental use of friends in the context of the Questioning/stalking behavior but our current knowledge of friends’ influence on one’s mate choice is very limited and would deserve systematic research (Shuangyue Zhang & Kline, 2009). Moreover, prior studies (e.g., Lindová et al., 2020) show that specific harsh behavioral patterns, such as coercive behavior or withdrawal of parental support (Rodrigues et al., 2017), affect relationship quality. Because parents and their offspring maintain a mutual relationship throughout life (Zarit & Eggebeen, 2002), future studies should explore the impact of various behavioral actions on relationship quality, either between the parents and offspring or between the offspring and their partners.

### Conclusions

To conclude, our study showed that even in an individualistic society, such as the Czech one (Hofstede, 2010), parents tend to interfere in their offspring’s relationships surprisingly during the offspring’s adulthood and in some cases even when the offspring already have their own family (some of the offspring in our sample already had children). Importantly, interfering parental behavior targets not only the offspring but also the offspring’s partner and the couple as a whole. In the last-named case, parental behavior tends to be the least disruptive because faced with the defensive behavior or dissent of the couple as a unit, parents’ negative interference is less likely to succeed. Overall, parents’ actions varied from supportive to disruptive ones, though most frequently reported were behaviors which were ambivalent or supportive. This may be first of all due to the design of our study: our findings are based on actual interactions which are much less likely to include extreme actions such as those reported in earlier studies that were based on hypothetical scenarios (Apostolou, 2013, 2015, 2017). Secondly, the likelihood of engaging in extreme behavioral patterns might slowly decrease with the offspring’s age because with age, the relationship between parents and offspring tends to improve in a linear fashion (Tighe et al., 2016) and parental influence wanes as offspring’s independence increases (Apostolou, 2013). Most reports on parent–offspring interactions came up with similar sets of actions. Differences among studies may be due to insufficient information about the behavior of the other member of the parent–offspring dyad, recall bias (Bell & Bell, 2018), or the result of self-enhancement tendencies (Krueger, 1998). Our study also highlights the need for considering the individuals concerned in a non-reductive fashion (Fišerová et al., 2021), in this case the inclusion of both members of the parent–offspring dyad to limit self-reporting bias, which could in cases such as those studied here take the form of social desirability bias (Bergen & Labonté, 2020), and to limit the effect of emotional state at the time of data collection (Cohen et al., 1988).

## Supplementary Information

Below is the link to the electronic supplementary material.

Supplementary file1 (PDF 143 KB)
