# Supplementary material for: Parental Interference in Offspring’s Mate Choice: Sets of Actions and Counteractions Based on Both Perspectives
Source: Arch Sex Behav. 2023 Feb 17;52(6):2447–63. doi: 10.1007/s10508-023-02544-3 (PMC9936925; doi:10.1007/s10508-023-02544-3)
Supplement: Supplementary file 1 — Supplementary file1 (PDF 143 KB) [file 10508_2023_2544_MOESM1_ESM.pdf]

Supplementary material

Parental interference in offspring's mate choice: Sets of actions and counteractions based on both perspectives

Archives of Sexual Behavior

[authors blinded for review]

Interview

I. Questions for the offspring

1) How would you describe your current partner?

2) What do you value in your partner?

3) What do you dislike about her/him?

4) Imagine you do not have a partner. What kind of partner would you definitely do not want?

5) What should he/she be like?

6) What kind of partner would your parents not approve of?

7) From what reactions (on the part of your parents) could you tell their opinion?

8) How do you think they would react if you were in a relationship with such partner?

9) Do you talk to your parents about your partner (previous partners)?

10) What kind of ideal partner would your parents imagine for you?

Mate choice – beginnings of the relationship (when parents found out about the relationship)

11) How long after you started dating did you tell your parents about your current relationship?

12) How long after you started dating did you introduce your partner to your parents? On what occasion was it, in what context?

13) What did they say about her/him?

14) Did they let you know what they thought of him/her? If so, how in particular?

15) At the beginning of your relationship, did they try to interfere in your relationship in any way to influence it (e.g., support you, talk you out of it)? If so, how in particular?

Current relationship

16) You have been in a relationship with your partner for XX months. How did your parents' attitude towards your partnership evolve? Is it now the same as before (when they found out about the relationship)?

17) Nowadays, are your parents letting you know what they think of your partner/relationship? If so, how in particular?

18) Are they trying to actively interfere in the relationship and influence it (support you, talk you out of it)? How in particular?

19) How do you perceive the fact that your parents do/do not interfere in your relationship?

a) How do you find it on the level of you and them, does it affect your relationship with your parents? If so, how?

b) Does it affect your relationship with your partner? If so, how?

c) How does your partner view it? Is he/she aware of your parents' interference in your relationship or absence thereof?

20) Is your parents' opinion (consent/disagreement) regarding your partnership important to you?

21) What do you think should be the ideal behaviour/role of parents in relation to their offspring's partnership?

22) Does your real situation correspond to this?

Previous partners

23) How did your parents view your former long-term partners?

24) What exactly did they value in them and what did they mind about them?

25) Did your parents try to actively interfere in your past relationships and influence them?

26) Has it ever happened to you that you did not start the relationship because of your parents or that you ended it because of them?

27) Would you like to add something on this subject that I did not ask about?

II. Questions for the parent

1) How would you describe your offspring's partner?

2) What do you value about your offspring's partner?

3) What do you not like about her/him?

4) Imagine that your offspring does not have a partner. What kind of partner would you not approve of as a parent?

5) How would you react if your offspring had a relationship with such a partner?

6) On the contrary, what should your offspring's partner be like?

7) Do you talk to your offspring about his/her partner (previous partners)?

Mate choice – beginnings of the relationship (when the parents found out about the relationship)

8) How long after they started dating did find out about your offspring's relationship?

9) After how long and in what context did you meet your offspring's partner?

10) What were your first impressions of her/him?

11) Did you let your offspring know what you think of his/her partner? If so, how in particular?

12) Did you try to interfere in the relationship and influence it (support your offspring, talk him/her out of it) in any way at first? If so, how in particular?

Current relationship

13) Your offspring has been in a relationship for XX months. How did your attitude towards this partnership evolve? Is it now the same as at the beginning (when you found out about the relationship)?

14) Are you now letting your offspring know what you think of her/his partner or the relationship? If so, how in particular?

15) Are you trying to actively interfere in the relationship and influence it (support your offspring, talk him/her out of it)? If so, how in particular?

16) How do you view the fact that you are/are not interfering in your offspring's relationship?

a) Does it affect your relationship with the offspring? If so, how?

b) Does it affect your relationship with your offspring's partner? If so, how?

c) Does it affect your relationship with your partner? If so, how?

d) Is your partner aware that you interfere/do not interfere in your offspring’s relationship?

17) Is expressing your opinion (consent/disagreement) about the offspring’s partnership important to you?

18) What do you think the ideal behaviour/role of parents towards their offspring’s partnership should look like?

19) Does your real situation correspond to this?

20) As a parent, what was your opinion of your offspring’s former long-term partners?

21) What specifically did you appreciate about them and what did you mind about them?

22) Have you tried to actively interfere in your offspring’s previous relationships and influence them?

23) Has it ever happened that your offspring did not start or ended his/her relationship because of you?

24) Would you like to add something related to the subject that I did not ask?

Note. Questions targeting the offspring n. 1 to 8, 10, and 21; and questions targeting the parent n. 1 to 6 and 18 were not involved in the analysis of this paper. However, responses including actions or counteractions were comprised into the analytical process.
